# KRAS^G12^ mutant induces the release of the WSTF/NRG3 complex, and contributes to an oncogenic paracrine signaling pathway

**DOI:** 10.18632/oncotarget.10625

**Published:** 2016-07-16

**Authors:** Yan Liu, Shu-Qing Wang, Yue-Hong Long, Su Chen, Yu-Feng Li, Jing-Hua Zhang

**Affiliations:** ^1^ College of Life Science, North China University of Science and Technology, Tangshan, 063000, China; ^2^ Cancer Institute, Tangshan People's Hospital, Tangshan, 063001, China; ^3^ Hospital of the North China University of Science and Technology, Tangshan, 063000, China; ^4^ Department of Nephrology, Affiliated Kailuan General Hospital of North China University of Science and Technology, Tangshan, 063000, China; ^5^ School of Life Sciences and Technology, Department of Breast Surgery of Yangpu Hospital, Research Center for Translational Medicine at East Hospital, Tongji University, Shanghai, 200092, China

**Keywords:** RAS, WSTF, NRG3, paracrine signaling

## Abstract

It remains unclear how the signals of mutant KRAS^G12^ in the transformed cells spread to the surrounding non-mutated cells and changes the microenvironment to promote tumor formation. We identified that Williams–Beuren syndrome transcription factor (WSTF), a non-secretory protein, was released in complex with secretory protein-neuregulin-3 (NRG3). The KRAS^G12^ mutant activates the transcription of *NRG3*. The WSTF/NRG3 in extracellular space could activate oncogenic pathways in normal colon cells carrying wild type KRAS and endow them with the ability to express NRG3 and release WSTF/NRG3. Extracellular WSTF/NRG3 promotes the formation of colon tumors. Blockade of extracellular WSTF could restore cetuximab sensitivity of colon cancer cells with mutant KRAS. The appearance of WSTF/NRG3 in serum and urine correlates with a colon tumor carrying a KRAS^G12^ mutant. In summary, our demonstration provides a new pathway to our understanding of the biological development of complex diseases.

## INTRODUCTION

Mutated *KRAS* oncogene was identified in multiple types of cancers, such as colon cancer (35%–45%) [1, 2]. The expression of mutant RAS glycine 12 to valine (G12V), promotes tumor initiation by activating different effectors, including Raf, phosphoinositide 3-kinase (PI3K), and Ras-like (Ral) guanine nucleotide exchange factor (RalGEF) [3]. Activation of the RAS pathway can induce tumorigenic phenotypes through exploiting autocrine and paracrine signaling mechanisms in normal or tumor cell lines [4, 5]. The existing data reveal that the balance of autocrine and paracrine signaling is important to tumorigenicity induced by mutations in *RAS* genes. However, how this balance becomes disrupted is still not clear.

Here, we demonstrate that *KRASG12* induces “secretion” of intracellular non-secretory protein Williams–Beuren syndrome transcription factor (WSTF) through the activation of silenced (*neuregulin*-*3*) *NRG3* in colon cells. WSTF is released into the extracellular space through forming a complex with secretory NRG3. The WSTF/NRG3 complex mediated cell–cell communication leads to the activation of oncogenic pathways of the surrounding normal colon cells and promotes the formation of colon tumor.

## RESULTS

### KRAS^G12V^ mutation results in the release of WSTF

**Figure 1:**
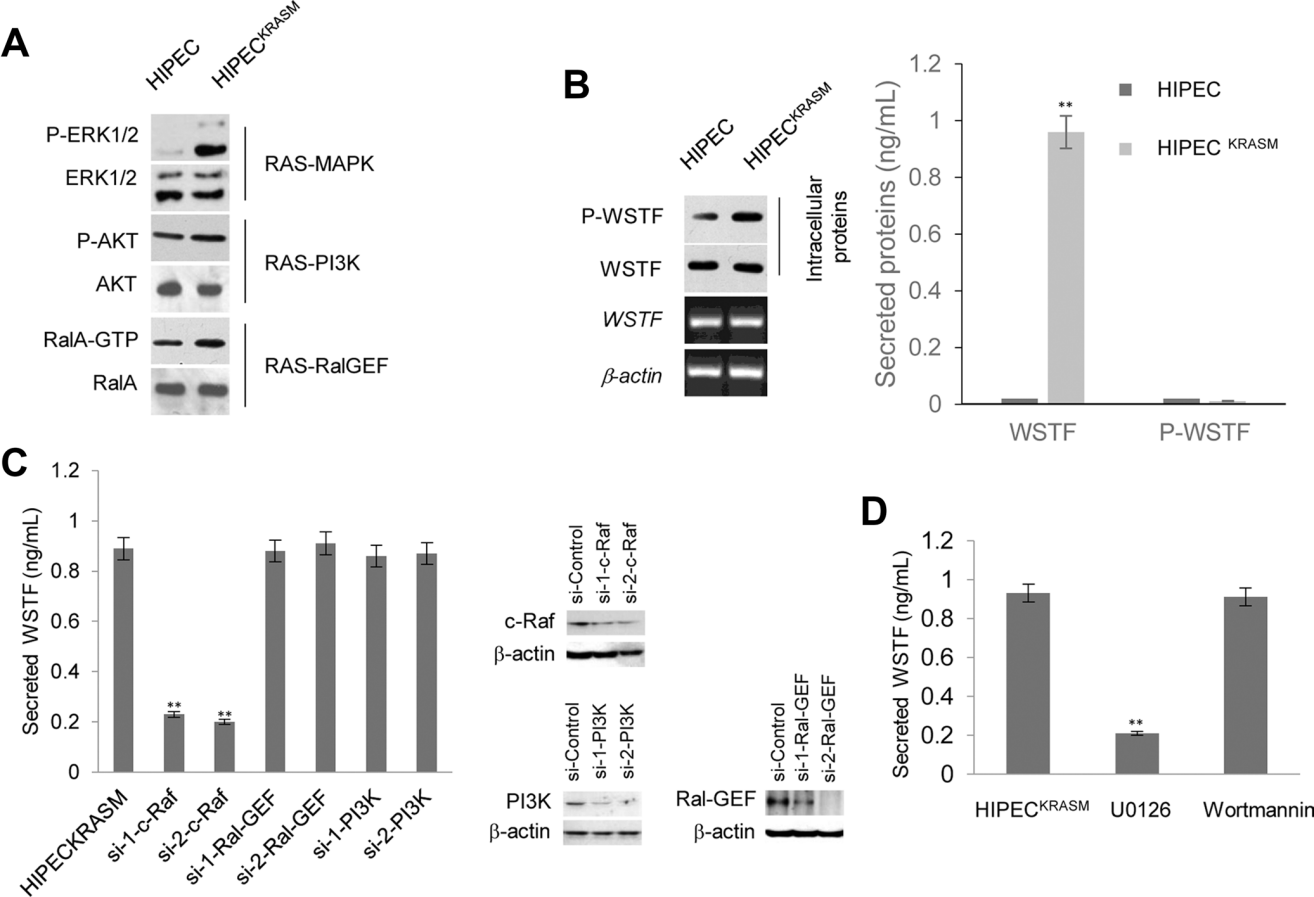
Release of WSTF was induced by KRAS^G12V^ in colon cells (**A**) We constructed the H-Ras^G12V^ expression vectors to preferentially activate the Ras pathways. The levels of P-ERK1/2, P-AKT and RalA-GTP were detected with specific antibodies to examine the activities of corresponding pathways. (**B**) Media containing WSTF and P-WSTF was detected using ELISA. Levels of intracellular P-WSTF, total WSTF protein and mRNA were detected as controls. (**C**) Different small interfering RNAs were transfected into HIPEC^KRASM^ cells for 48 h. Two specific siRNAs for each gene were implicated in experiments. The secreted WSTF was detected by ELISA assay. (**D**) The media of HIPEC^KRASM^ cells, which was cultured with U0126 (10 *μ*M) or Wortmannin (10 *μ*M) for 20 h, were tested by ELISA. Non-treated HIPEC^KRASM^ cells were used as control.

A non-transformed human intestinal primary epithelial cell (HIPEC) line was established according to the method described in Panjia's report [6], in which the wild type (WT) *KRAS* was detected by direct sequencing. A stable HIPEC line, which was introduced with *KRASG12V* mutation through transfecting pEGFP-N1-human-H-Ras^G12V^ plasmid, was designated as HIPEC^KRASM^. In HIPEC^KRASM^, the activity of the RAS–mitogen-activated protein kinase (MAPK) pathway was obviously enhanced with higher levels of phosphorylated extracellular signal-regulated kinase 1/2 (P-ERK1/2) compared with normal HIPEC cells (Figure 1A). The RAS-PI3K and -RalGEF pathways were activated to different levels (Figure 1A).

With the intention to examine secreted proteins of HIPECs following the introduction of *KRASG12V*, we detected WSTF in the cell media unexpectedly, which was designed as a negative control (Figure 1B). WSTF locates mainly in the nucleus and is involved in chromatin assembly [7, 8]. No known signal peptide or transmembrane domain sequences of secretory protein were identified in WSTF [8]. Interestingly, P-WSTF (phosphorylated at Ser 158) was markedly increased intracellularly in HIPECs^KRASM^ compared with original HIPECs, whereas no P-WSTF was detectable in either media (Figure 1B). We speculate that WSTF in the media may be released actively from the living HIPECs^KRASM^, but not from fragmented cells.

To address whether the release of WSTF was induced by RAS signal, specific small interfering RNAs (siRNAs) were transfected into HIPECs^KRASM^ to block MAPK, Ral-GEF, or PI3K pathway. As shown in Figure 1C, WSTF releasing was strongly decreased following the blockade of MAPK pathway by specific c-Raf siRNA, but not the blockade of the other two pathways. To further confirm this result, specific inhibitors were used to interrupt the transduction of MAPK or PI3K signal. As shown in Figure 1D, the release of WSTF was downregulated by MAPK pathway inhibitor U0126, but not by PI3K pathway inhibitor Wortmannin.

### Secretion of WSTF was not activated by exocytosis system

**Figure 2:**
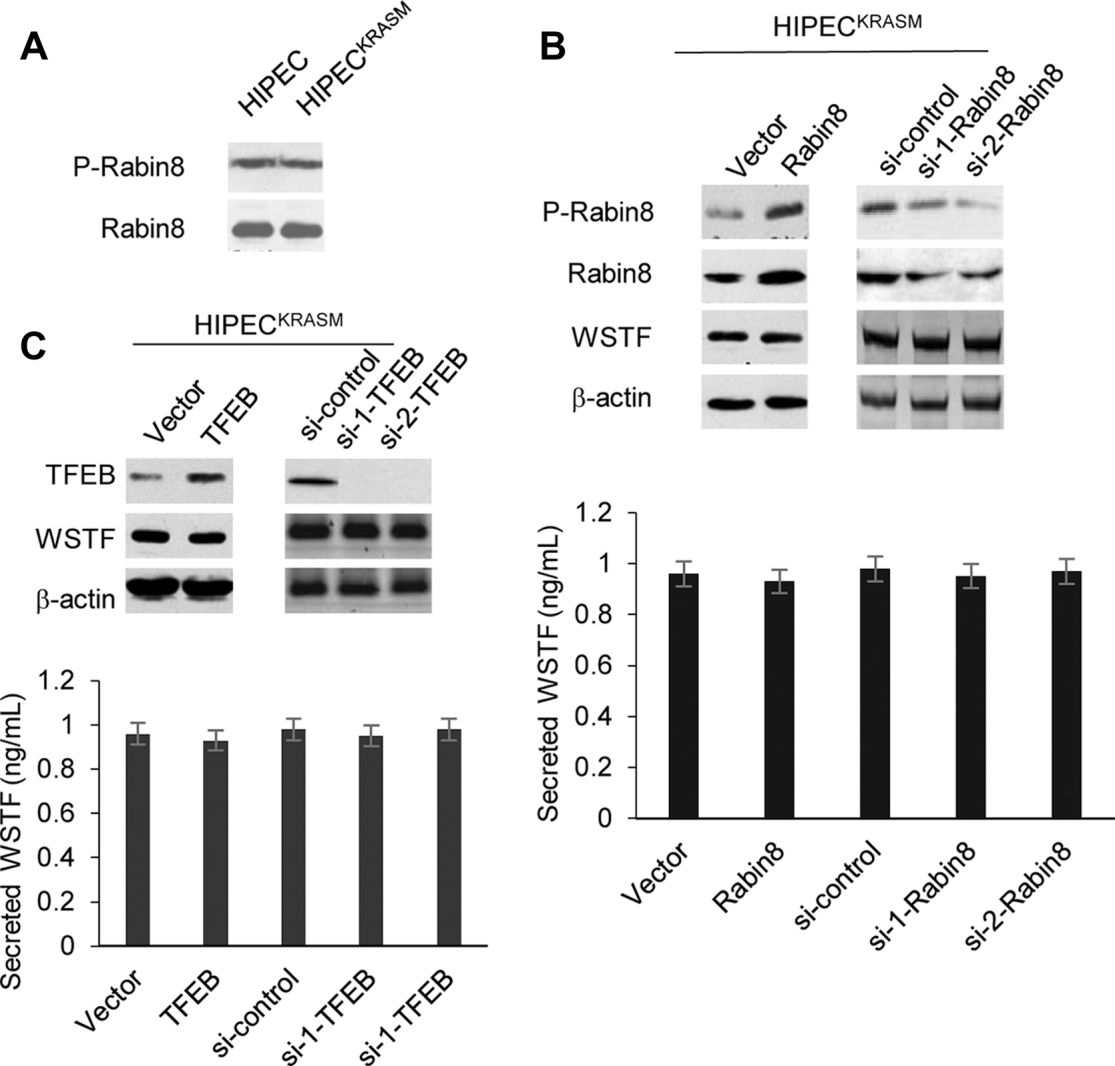
Rabin8 and TEFB do not stimulate the release of WSTF (**A**) The whole cell lysates were collected from the indicated cells. The level of P-Rabin8 was analyzed using WB and total Rabin8 protein was tested as control. (**B** and **C**) Rabin8 or TFEB expressing plasmid (1 μg, 2 μg or 4 μg) and specific siRNAs were transfected into the HIPEC^KRASM^ cells. The empty vector and scrambled siRNA were used as controls. 48 hours after transfection, the cell lysates and media were collected to perform WB and ELISA.

Next, we wanted to explore the mechanism regulating the release of intracellular protein WSTF. Rabin8 is an important factor of the exocytosis system that stimulates vesicular trafficking to the plasma membrane, and is known to be activated by MAPK pathway [9]. Therefore, we investigated the phosphorylation level of Rabin8 in response to KRAS mutation, however, no obvious changes were detected (Figure 2A). Further, knock-down or overexpression of Rabin8 had no greatly effect on the WSTF levels in media (Figure 2B), so the release of WSTF does not appear to be due to Rabin8.

Next, to test the possibility of the involvement of lysosomal exocytosis, we checked the key component of this system, transcription factor EB (TFEB) [10]. The TFEB protein was increased or decreased with the introduction of expressing plasmid or RNAi. However, the level of WSTF in media did not fluctuate according to the change of TFEB level as shown in Figure 2C.

### KRAS mutation activates the NRG3 protein expression to transport WSTF

**Figure 3:**
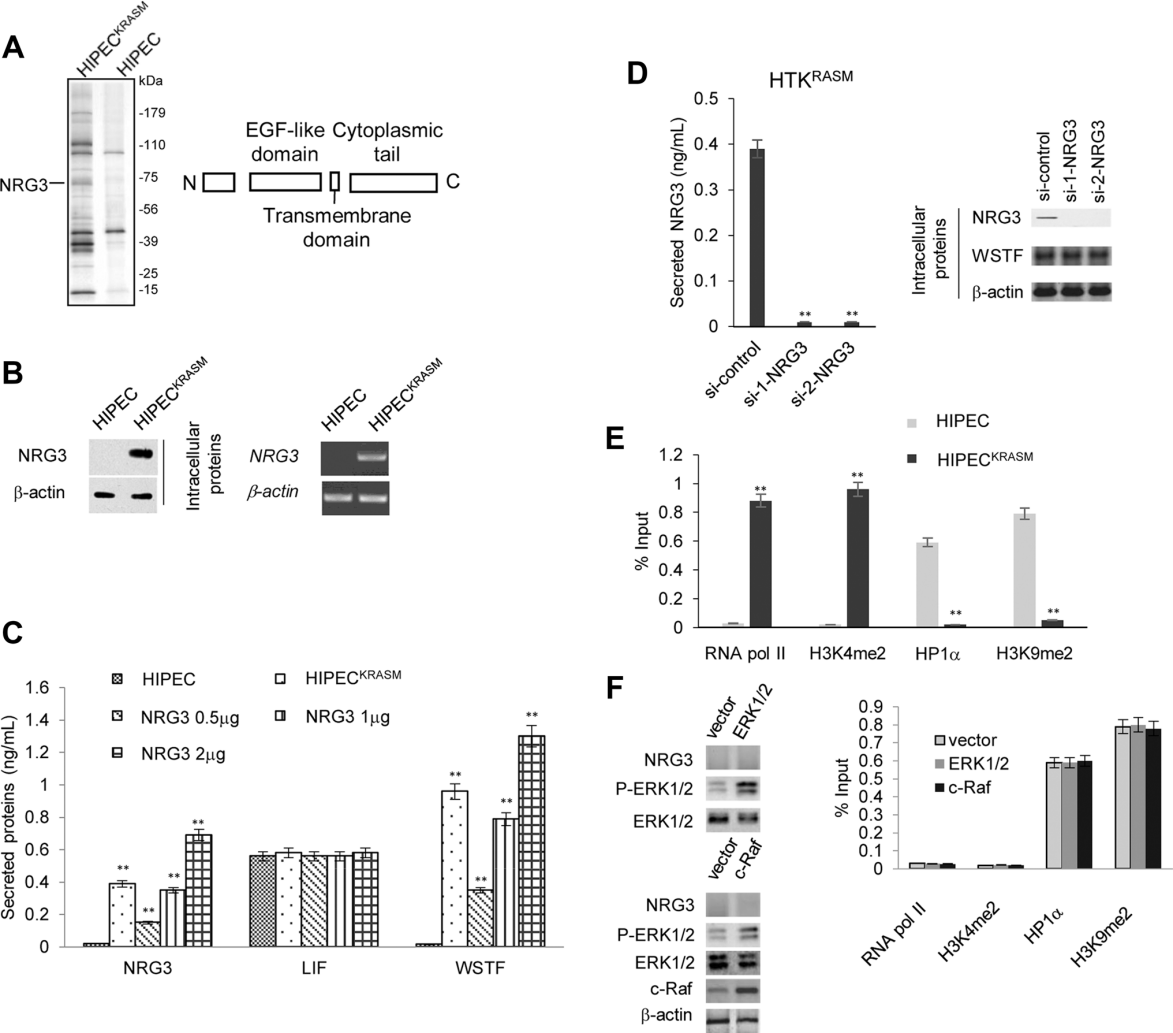
WSTF release was mediated by NRG3 following KRAS^G12V^ expression (**A**) Cellular extracts from HIPEC and HIPEC^KRASM^ cells stably expressing FLAG-WSTF were immunopurified with anti-FLAG affinity columns and eluted with FLAG peptide. The eluates were resolved by SDS-PAGE and protein bands were retrieved and analyzed by mass spectrometry. The main functional domains of NRG3 were diagramed. (**B**) Endogenous mRNA and protein level of NRG3 were assayed by real time RT-PCR and WB. RT-PCR analysis was performed on 0.25–0.5 *μ*g total RNA using primers specific for *NRG3* for 35 PCR cycles. (**C**) Different amount of NRG3-expressing plasmids were transfected into WT HIPECs. NRG3, WSTF and LIF in the media were detected by ELISA. WT HIPECs was used as control. (**D**) HIPEC^KRASM^ cells were transfected with control or *NRG3*-specific siRNA and then ELISA were performed with the media. (**E**) ChIP assay was performed to detect the proteins that bind at the promoter region of *NRG3*. (**F**) HIPECs were transfected with ERK1/2 or c-Raf expression plasmid followed by western blot to detect the level of P-ERK1/2 and NRG3. ChIP assay was performed to measure the recruitment active or inactive markers at the *NRG3* promoter region.

We then wondered whether WSTF was carried out of the cell by an ectopic secretory protein. To confirm this possibility, FLAG-WSTF was stably expressed in wild-type HIPEC and HIPEC^KRASM^ cells and then subjected to affinity purification and mass spectrometry. NRG3, one of the neuregulin family members was identified to associate with WSTF following the introduction of KRAS^G12V^ (Figure 3A). Human NRG3 has been identified in the developing breast and central nervous system, but not in other normal tissues [11, 12]. Ectopic expression of NRG3 was detected in a proportion of primary breast cancer biopsies (42%) with undefined function [13–15].

Further experiments were performed to test whether NRG3 in HIPECs^KRASM^ is induced by KRAS mutation. NRG3 was not detected intracellularly and extracellularly under the circumstances of WT-KRAS, whereas the KRAS^G12V^ mutation resulted in the expression and secretion of NRG3 (Figure 3B–3C).

Besides the ectopic expression and secretion of NRG3, no significant changes of leukemia inhibitory factor (LIF) secretion were observed, which was induced by the RAS-MAPK pathway activation and was sufficient to induce growth arrest and differentiation of surrounding cells (Figure 3C). These results demonstrate that the balance of paracrine signaling that is activated by the RAS-MAPK pathway may be broken in KRAS^G12^ mutation induced colon cancer.

Next, we sought to observe whether overexpression of NRG3 is sufficient to induce the secretion of WSTF in the absence of activated Ras mutation in HIPECs. Different amount of NRG3-expressing plasmids were transfected into wild-type HIPEC cells. As expected, secretory WSTF was detected in the media with NRG3 proteins (Figure 3C). Moreover, WSTF was undetectable in the media of HIPECs^KRASM^ following NRG3 knock-down (Figure 3D).

To understand the possible reason of *NRG3* silencing in colon cells, we analyzed the proteins binding at the *NRG3* promoter through Chromatin Immunoprecipitation (ChIP). In HIPECs, active markers of transcription, such as RNA pol II and histone 3 lysine 4 dimethylation (H3K4me2), were absent, whereas markers of transcriptional repression, such as heterochromatin protein 1α (Hp1α) and H3K9me2, were detected at the *NRG3* promoter region (Figure 3E). Conversely, in HIPECs^KRASM^ the promoter region of *NRG3* showed signs of active transcription (Figure 3E). We surmise that KRAS^G12V^ induced changes in histone modifications and Hp1α alter the chromatin structure and then activate the transcription of *NRG3*, which is in agreement with similar RAS signaling events that we have previously identified [3].

To test whether the increased P-ERK1/2 is sufficient enough to induce NRG3 expression without KRAS^G12^ mutation, expression plasmid for ERK1/2, or c-Raf was transfected into HIPECs. NRG3 expression was not detected following the upregulation of P-ERK1/2, and the recruitment of RNA pol II, H3K4me2, Hp1α and H3K9me2 at the *NRG3* promoter region were not strongly changed as measured by ChIP assay (Figure 3F). This result revealed that P-ERK1/2 signaling alone is insufficient to initiate the expression of NRG3.

### NRG3 directly binds WSTF

**Figure 4:**
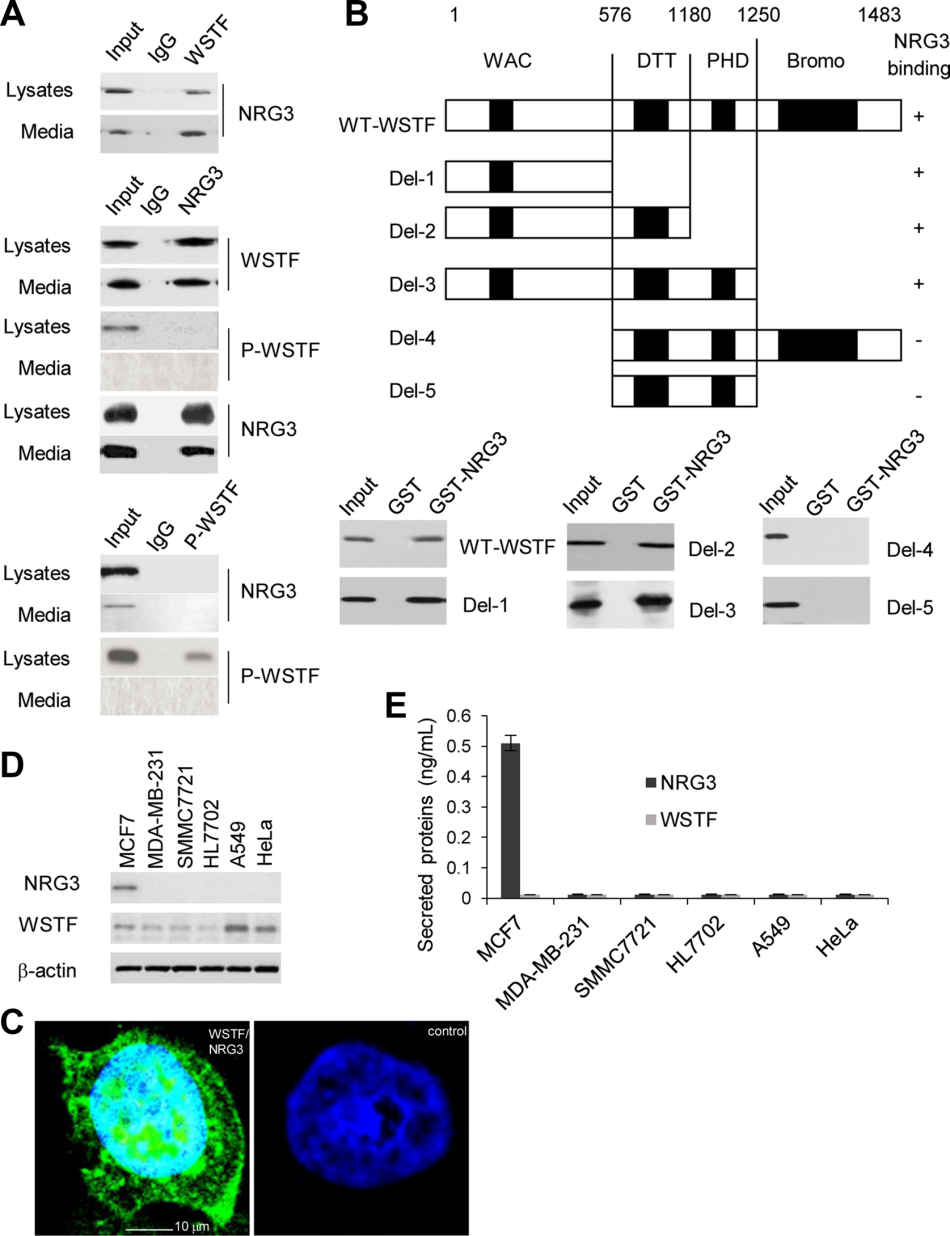
NRG3 directly associates with WSTF *in vivo* and *in vito* (**A**) HIPEC^KRASM^ cell lysates or media were immunoprecipitated with antibodies against WSTF or P-WSTF, or immunoprecipitated with antibody against NRG3. (**B**) GST pull-down assay were performed with NRG3-GST and WSTF-Myc to determine the domain of WSTF that is critical for its association with NRG3. (**C**) *In situ* PLA was performed using rabbit anti-WSTF and mouse anti-NRG3 antibody. Nuclei were indicated as DAPI staining. Control was stained with non-immune rabbit- and mouse serum. The cells were observed by confocal fluorescence microscopy (× 400). (**D** and **E**) NRG3 and WSTF were detected in different cancer cell lines.

To confirm the association between NRG3 and WSTF, HIPEC^KRASM^ cells lysates or media were collected and co-immunoprecipitation were performed with antibody against WSTF followed by immunoblotting with antibody against NRG3. The data indicated that NRG3 binds WSTF (Figure 4A, upper panel). Reciprocal co-immunoprecipitation with antibody against NRG3 and immunoblotting with antibody against WSTF also showed that WSTF binds NRG3 (Figure 4A, middle panel). No co-immunoprecipitation of NRG3 and P-WSTF was detected (Figure 4A, middle panel and lower panel).

Furthermore, to investigate the regions of WSTF binding with NRG3, glutathione S-transferase (GST) pull-down was conducted with NRG3-GST and wild type or serial truncated mutants of Myc-tagged WSTF, which were produced as previously reported [7]. The results revealed that WSTF could bind NRG3 directly and the region around amino acids (aa) 1–576 of WSTF is critical for binding with NRG3 (Figure 4B). To detect the binding of endogenous WSTF to NRG3 *in vivo*, WSTF/NRG3 complex in HIPEC^KRASM^ cells was detected by *in situ* proximity ligation assays (PLA). As shown in Figure 4C, *in situ* PLA signals were detected as green dots.

We were interested to investigate the binding of NRG3 with WSTF in other human cancer cell lines and found that the association between NRG3 and WSTF could be unique in colon cells. As shown in Figure 4D, NRG3 only was observed in MCF7 cells; nevertheless, NRG3 and WSTF were not detected in the MCF7 media (Figure 4E).

### Secreted WSTF/NRG3 activates the release of WSTF/NRG3 from normal colon cells and increases the activities of oncogenic pathways

**Figure 5:**
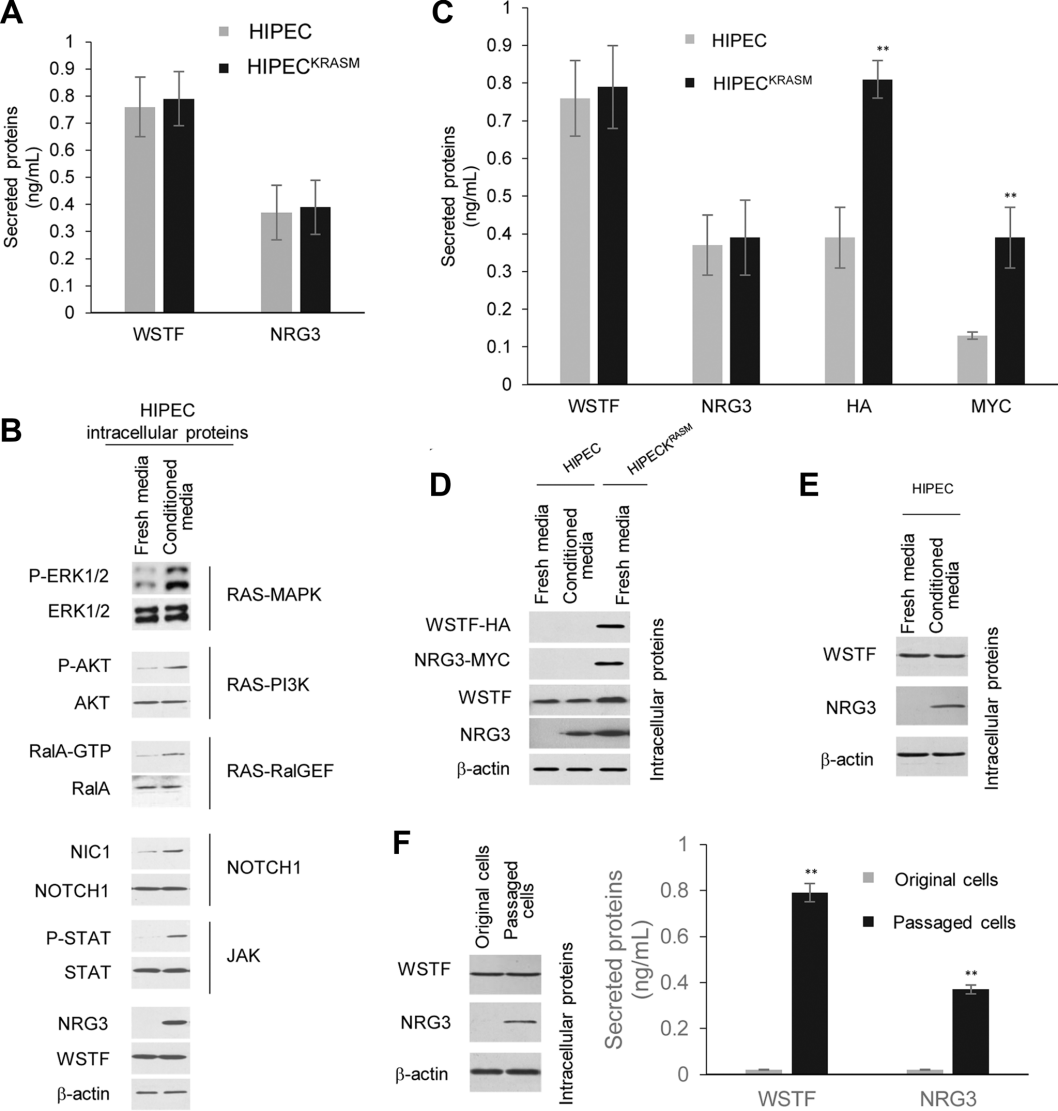
Secreted WSTF/NRG3 stimulates the release of WSTF/NRG3 in normal colon cells and enhances the RAS/NOTCH1/JAK pathway activities (**A**) ELISA assay were used to test the levels of WSTF/NRG3 in the media. (**B**) Several signaling pathways were measured through western blot with the indicated antibodies. The HIPECs cultured with fresh media were used as control. (**C**) The same number of HIPECs^KRASM^ and HIPECs were seeded in 6-well plates and cultured with normal or conditioned media respectively. After 24 hours culture, different media were collected for ELISA assay with the indicated tags or WSTF or NRG3 antibodies. (**D**) Cell lysate were collected from normal colon cells following cultured with conditioned media. WSTF/NRG3 and tags were detected with the antibodies indicated. The HIPECs and HIPECs^KRASM^ cultured with fresh media were used as controls. (**E**) Normal colon cells was cultured with fresh media which contain equal amount of WSTF and NRG3 (indicated as conditioned media). The cell lysate were collected to perform immunoblot assay. (**F**) HIPECs were cultured with conditioned media (half fresh and half from HIPECs^KRASM^) for 48 hours. Then the media was removed and the cells were washed with PBS and further cultured with fresh normal media for several passages in the next month, which were indicated as passaged cells. The HIPECs routinely cultured with fresh normal media were indicated as original cells.

As NRG3 is not expressed in normal HIPECs, we wanted to explore the effects of WSTF/NRG3 release as a consequence of KRAS^G12V^. HIPECs^KRASM^ were seeded in 6-well plates and after overnight incubation, the media was collected and half was analyzed for WSTF/NRG3, while the other half was mixed with equivalent fresh media. This conditioned media was then used to culture wild-type HIPEC cells that were seeded in a 6-well plate for 20 hours followed by WSTF/NRG3 secretion analysis. The WSTF or NRG3 concentration in the media from wild-type HIPECs was almost equal to that in the HIPECs^KRASM^ media (Figure 5A). The recovery of WSTF/NRG3 concentration after dilution with equal amount fresh media suggest the normal HIPECs acquire the ability to release WSTF/NRG3 after cultured with the conditioned media.

The cell lysate from the wild-type HIPEC cells treated with conditioned media were also analyzed. The results demonstrated that the activities of RAS, NOTCH1 and JAK pathways become much higher in wild-type HIPECs following culture with the conditioned media (Figure 5B). Importantly, the NRG3 expression was observed in the wild-type HIPECs cultured with the conditioned media (Figure 5B). These results indicate that the WSTF/NRG3 released from KRAS^G12V^ mutant cells initiates the release of WSTF/NRG3 from normal colon cells and this function could be a consequence of enhancement of the oncogenic pathway activity.

To confirm the function of WSTF/NRG3 the media from HIPECs^KRASM^, which stably express HA-WSTF and MYC-NRG3, was collected and used to culture wild-type HIPECs with equal volume of fresh normal media. Then the media was collected for an ELISA assay. Endogenous and exogenous WSTF/NRG3 was both detected (Figure 5C). HA-/MYC tagged WSTF/NRG3 was diluted after mixing with fresh media, whereas the total amount of WSTF/NRG3 was almost the same in the final media compared to the original media. Moreover, western blot was applied to analyze the endogenous levels of WSTF/NRG3 in the wild-type HIPECs that were cultured with the conditioned media. Endogenous WSTF/NRG3 was detected without any tag signal (Figure 5D). Same result was observed through *in situ* PLA assay (negative figures were not shown). These results indicate that WSTF/NRG3 in the media may initiate the release of endogenous WSTF/NRG3 from the wild-type colon cells through specific receptor pathways rather than entering into the cells. This was supported by the fact that a western blot experiment demonstrated that HIPECs express endogenous NRG3 after cultured with fresh media, which contains an equal amount of WSTF and NRG3 (Figure 5E).

Furthermore, after culturing with the media containing WSTF/NRG3 for 48 hours, the conditioned media was removed and the wild-type HIPEC cells were washed with PBS before culturing with fresh normal media for several passages. Surprisingly, we noticed the daughter generations of HIPECs inherit the capability to produce and release WSTF/NRG3 after culturing with the conditioned media (Figure 5F).

### Extracellular WSTF/NRG3 promotes cancer formation

**Figure 6:**
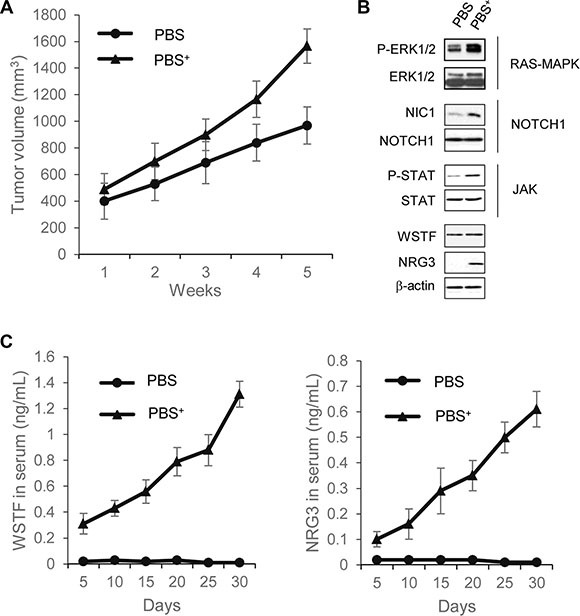
Extracellular WSTF and NRG3 promote tumor formation in mouse (**A**) Tumors were measured weekly with Vernier calipers, and volume was calculated using the formula π/6 × length × width^2^. Each point represents the mean ± S.D. for different animal measurements (*n* = 6). (**B**) The levels of the indicated proteins extracted from xenograft tumor were detected by western blotting. (**C**) Serum WSTF and NRG3 were measured through ELISA.

To further explore the role of WSTF/NRG3 release in tumorigenesis *in vivo*, tumor formation experiments were performed. SW48 cells (human colon cancer cell line containing WT KRAS) in PBS or PBS containing WSTF/NRG3 proteins (PBS^+^) were injected into 6- to 8-week-old female BALB/C mice. Measurements of the tumor indicated that tumors of the PBS^+^ group grew significantly faster than PBS group (Figure 6A).

To investigate the underlying mechanisms of this effect, the tumor cells were collected for further analyses of signaling pathways and WSTF/NRG3 expression. The RAS-MAPK, NOTCH1 and JAK pathways had much increased activity in PBS^+^ group in comparison to PBS group. The NRG3 protein expression was also detected in PBS^+^ group, but not PBS group. Collectively, the results reveal that WSTF/NRG3 stimulates the growth of tumors, and this stimulation could be related with activation of oncogenic pathways (Figure 6B).

We next sought to explore whether secreted WSTF/NRG3 could be detected in serum. The serum samples of mice were collected once per day for 5 days during the course of the experiment followed by ELISA assays. From the fifth day after injection, WSTF and NRG3 could be detected in serum samples of the PBS^+^ group and the levels were increased along with tumor growth (Figure 6C). This result reveals that the SW48 cells acquire the ability to release WSTF/NRG3 after injection with the protein mixture. It is noteworthy that the appearance of WSTF and NRG3 in serum was earlier than any detectable tumor mass.

### Extracellular WSTF/NRG3 could be developed as a diagnostic marker

**Figure 7:**
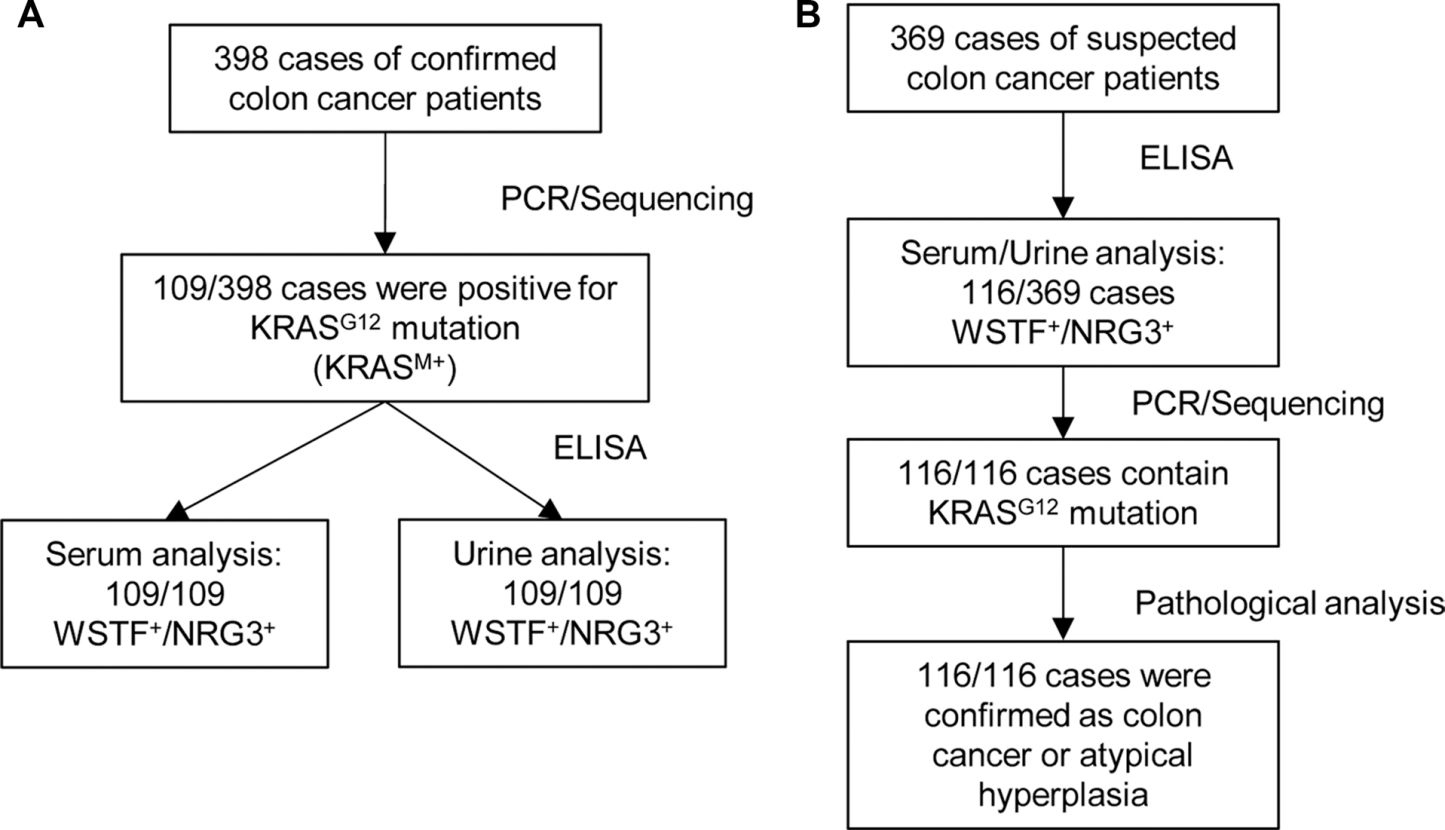
Extracellular WSTF and NRG3 could be promising diagnostic markers (**A** and **B**) Total RNA were extracted from fresh tumor tissues and the samples were tested by PCR and sequence analysis. The serum and urine samples were collected as described in Materials and Methods and further tested using ELISA.

Given the results that serum WSTF/NRG3 correlates with tumor formation, we sought to explore whether detection of the serum and urine WSTF/NRG3 could be developed into a clinical diagnostic test. We collected serum and 24-hour urine samples of 398 cases of colon cancer patients. Mutation of KRAS at G12 (G12V 58%, G12D 41%, others 1%) was identified in 109 of the 398 cases (KRAS^M+^). Furthermore, ELISA assay indicated that all the 109 cases were WSTF/NRG3 double positive (WSTF^+^/NRG3^+^) in serum and urine (Figure 7A).

To confirm the relationship between KRAS mutation and WSTF/NRG3 secretion, the serum and urine samples of a separate cohort of 369 cases with suspected colon cancer were collected. WSTF^+^/NRG3^+^ of both serum and urine samples were identified in 116 out of the 369 patients. Furthermore, PCR and sequencing analysis were performed with the tumor tissue samples of the 116 patients after surgery. The results indicated that all the 116 cases contain KRAS mutation at G12 (G12V 61%, G12D 37%, others 2%). After pathological analysis, 100 of 116 were diagnosed as colon cancer and the other 16 were confirmed as atypical hyperplasia (Figure 7B).

### Blockade of WSTF could reverse the drug resistance associated with KRAS mutant

As we mentioned previously, the release of WSTF activates NOTCH1 and JAK, in addition to RAS pathways. These three pathways are all relevant to drug resistance [16]. Patients with colon cancer who receive the epidermal growth factor receptor (EGFR) targeted antibodies cetuximab or panitumumab usually develop resistance within several months of initiating therapy. The emergence of mutations in KRAS is associated with acquired resistance to EGFR blockade [17, 18].

**Figure 8:**
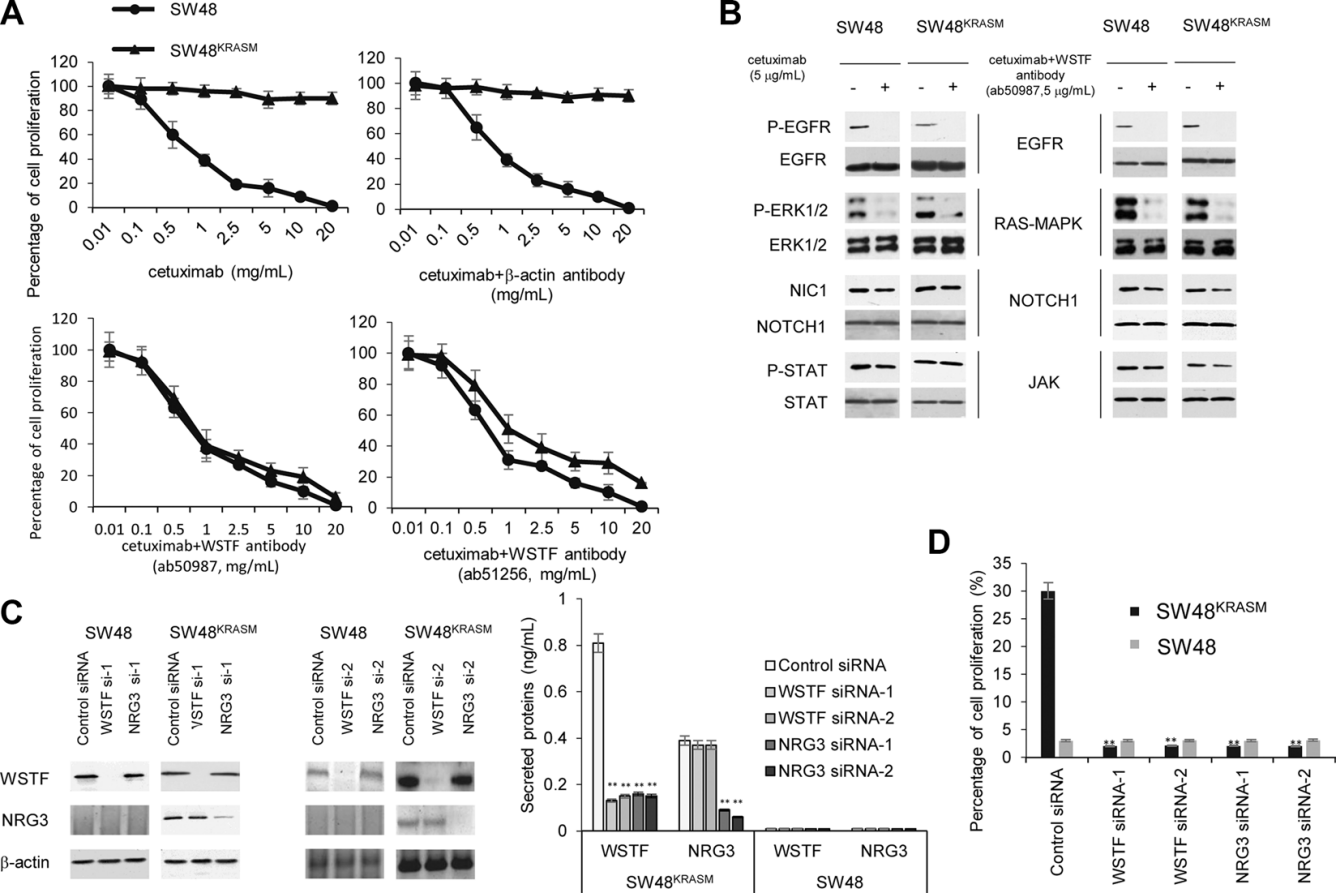
Blockade of WSTF could restore cetuximab sensitivity (**A**) SW48 and SW48^KRASM^ cells were treated with increasing concentrations of cetuximab with or without anti-WSTF antibodies for 48 hours and evaluated for proliferation by CCK-8. Cetuximab combining anti-β-actin antibody was used as control. (**B**) Cells were treated with cetuximab or combination of cetuximab with anti-WSTF antibodies for 48 hours. The cell lysates were collected for protein analyses with indicated antibodies. (**C** and **D**) *WSTF* or *NRG3* were knocked down by siRNAs. 24 hours later, medium were added with cetuximab (8 μg/mL) for 20 hours and then viable cells were calculated and plotted relative to untreated control. Intracellular and extracellular WSTF and NRG3 were tested using western blot and ELISA. SW48 cells were used as control.

To explore whether the blockade of WSTF was helpful in the treatment of EGFR drug resistance patients, we added cetuximab in combination with WSTF or β-actin antibodies to the media of SW48 or SW48^KRASM^ cells. As illustrated in Figure 8A, SW48^KRASM^ cells were not sensitive to cetuximab alone at doses up to 20 μg/mL The addition of WSTF antibodies (Abcam, ab50987 and ab51256) could inhibit the proliferation of SW48^KRASM^ cells, whereas the addition of a β-actin antibody (ab8226) did not have this effect (Figure 8A). Furthermore, western blot results reveal that cetuximab treatment efficiently inhibited the phosphorylation of EGFR and downstream pathways activation (Figure 8B), but not the ERK1/2, NOTCH1 and JAK pathways. However, the combination of cetuximab and WSTF antibody blocked these pathways remarkably in both SW48 and SW48^KRASM^ cells (Figure 8B).

Next, we investigated whether a reduction in WSTF expression and secretion could restore cetuximab sensitivity. Intracellular and extracellular WSTF were decreased with *WSTF* or *NRG3* siRNA (Figure 8C). As show in Figure 8D, decreasing WSTF expression and release, in combination with cetuximab treatment, significantly inhibited SW48^KRASM^ cell growth.

## DISCUSSION

Cells carrying KRAS mutations were only detected in a fraction of the tumor biopsies from resistant patients. One possibility is that a paracrine cross-talk driven by the resistant subpopulations may provide protection for surrounding sensitive cells that have wild type KRAS. Because colon cancer cells with wild type KRAS could be transformed by WSTF/NRG3, they would be not sensitive to the targeted inhibition of EGFR. Moreover, the WSTF/NRG3 complex not only provides protection for the adjacent cells, the adjacent cells release their own WSTF/NRG3 to further amplify this signal.

The event we demonstrate can potentially be applied beyond cancer to all human diseases containing gene mutations, even in tissue development. WSTF and NRG3 may be developed into diagnostic markers, although further analyses of more cases of patients are necessary. The problems still remaining are the following: (1) How WSTF and NRG3 enter into blood and urine? (2) Whether the release of WSTF/NRG3 induced by KRAS^G12^ mutations is tissue or cell-type specific. (3) How the complex of WSTF and NRG3 is formed. (4) Whether mutations occur more frequently in normal cells following the stimulation by WSTF/NRG3; and (5) whether all normal colon cells or just a subpopulation that expresses certain receptors could be affected by WSTF/NRG3.

## MATERIALS AND METHODS

### Cell Culture and treatments

SW48, MCF7, MDA-MB-231, SMMC7721, HL7702, A549 and HeLa cells were obtained from the Cell Culture Center of Peking Union Medical College, and were maintained in proper medium. Cell line authentication and validation by short tandem repeats was performed on 16 May 2013. Nontransformed human intestinal primary epithelial cell (HIPEC) line were established according previous report [6] (from a 35-year old male patient undergoing surgery at Kai-Luan General Hospital for ulcerative colitis) and cultured in F-12 medium (Gibco) containing epidermal growth factor (EGF) (5 ng/ml), transferrin (500 μg/ml), insulin (500 μg/ml), hydrocortisone (100 μg/ml), and retinoic acid (5 μg/ml). Experiments were performed according to the manufacturer's instructions and previous report [19]. All cell lines were routinely screened for the presence of mycoplasma (Mycoplasma Detection Kit, Roche Diagnostics).

### Ethics statement

All studies performed with human cancer specimen and mice were approved by the Ethics Committee and Animal Care Committee of North China University of Science and Technology, and informed consent was obtained from all patients.

### Reagents and antibodies

The mouse monoclonal antibodies against β-actin (#TA09), HA-tag (#TA06) and Myc (#TA01) were purchased from Zhongshan Golden Bridge Biothechnology Co. (Beijing, China). The rabbit polyclonal anti-AKT antibody (9272), rabbit monoclonal anti-phospho-Akt (Ser-473) (4058) antibody and rabbit polyclonal anti-H3 (9715) antibody were purchased from Cell Signaling Technology. Rabbit polyclonal anti-ERK1/2 antibody (ab17942), anti-phosphorylated ERK1/2 antibody (ab138482), anti-RalA antibody (ab68837), anti-EGFR antibody (ab191813), anti-EGFR (phospho S1070+S1071), anti-Rabin8 antibody (ab90732), anti-TFEB antibody (ab82143), anti-Notch1 antibody (ab52627), anti-activated Notch1 antibody (ab8925), anti-STAT3 (ab119352), anti-STAT3 (phospho S727), monoclonal anti-WSTF antibody (ab51256) and anti-NRG3 antibody (ab109256) were all purchased from Abcam Trading (Shanghai, China) Company Ltd. Anti-RalGEF antibody (SAB2104233) was purchased from SIGMA-ALDRICH (Shanghai, China). Anti-phosphorylated WSTF^S158^ antibody was a gift from Pro. Zhao-Bin Xing. Rabbit polyclonal anti-RNA pol II antibody (ab5408), goat polyclonal anti-Hp1αantibody (ab77256), rabbit polyclonal anti-H3K4me2 antibody (ab7766) and mouse monoclonal anti-H3K9me2 antibody (ab1220) were obtained from Abcam. Wortmannin (ab120148) and U0126 (ab120241) were all purchased from Abcam.

### Plasmid construction and siRNA

The coding regions of human *KRAS*, *WSTF* and *NRG3* were amplified from 293T cDNA by polymerase chain reaction (PCR). The PCR products were subcloned into HA-tag or Myc-tag vectors and sequenced. The *KRAS G12* mutant was constructed using the TaKaRa MutanBEST Kit (catalogue number R401), as recommend by the manufacturer. siRNAs were purchased from Shanghai GenePharma. Two specific siRNAs for each gene were implicated in experiments.

### DNA sequence analysis

DNA samples were obtained with Wizard SV genomic DNA Purification System (Promega). PCR amplifications were performed using 0.8 mmol/L each of the primers, 0.3 mmol/L deoxynucleotide triphosphates, 1× PCR reaction buffer, 6% DMSO, 1 ng/mL genomic DNA and 0.05 unit/mL Platinum Taq (Invitrogen/Life Technologies). PCR products were purified using AMPure (Agencourt Bioscience Corp.; Beckman Coulter S.p.A). Sequencing was performed by CapitalBio Corporation (Beijing, China).

### Co-immunoprecipitation analysis

Co-immunoprecipitation assay was performed as described previously [19]. Briefly, cells were suspended with buffer and fragmented by sonication. Then, the cell lysates were reacted with normal IgG or different antibodies. The complex reacted with Protein A/G agarose beads. Next, the beads were washed with buffer and the deposited proteins were freed by boiling.

### Tail-vein collection of blood

Alcohol was used initially as a vasodilator, but it should not be used on broken skin. The tip of tail will be aseptically prepared or wiped with alcohol prior to the tail-snip or excision. The tail will be nicked with the sterile scalpel blade (< 0.5 mm) and the blood will be collected in a sterile capillary tubes. Pressure will be applied to the tail to stop bleeding and the mouse will be returned to the cage. If bleeding persists, the nicked tail will be cauterized using a silver nitrate stick.

### Statistical analysis

The statistical analyses were performed using the Student's *t* test. A *p* value of 0.05 was considered significant (*, *p* < 0.05; **, *p* < 0.01).

### Proliferation assay

Cells were cultured in 24-well plates and were treated with different concentrations of cetuximab (0.01–20 μg/mL) alone or in combination with equal amount of WSTF antibody for 48 h. Cell proliferation was measured with Cell counting kit (CCK)-8. Results represent the median of three separate experiments.
